# Cost-Effectiveness of Telemedicine in Remote Orthopedic Consultations: Randomized Controlled Trial

**DOI:** 10.2196/11330

**Published:** 2019-02-19

**Authors:** Astrid Buvik, Trine S Bergmo, Einar Bugge, Arvid Smaabrekke, Tom Wilsgaard, Jan Abel Olsen

**Affiliations:** 1 Department of Orthopaedic Surgery University Hospital of North Norway Tromsø Norway; 2 Norwegian Centre for E-health Research University Hospital of North Norway Tromsø Norway; 3 Centre for Quality Improvement and Development University Hospital of North Norway Tromsø Norway; 4 Department of Community Medicine The University of Tromsø - The Arctic University of Norway Tromsø Norway; 5 Centre for Health Economics Monash University Melbourne, Victoria Australia

**Keywords:** telemedicine, orthopedics, videoconferencing, remote consultation, outpatients, randomized controlled trial, economic evaluation, cost-effectiveness analysis, QALY

## Abstract

**Background:**

Telemedicine consultations using real-time videoconferencing has the potential to improve access and quality of care, avoid patient travels, and reduce health care costs.

**Objective:**

The aim of this study was to examine the cost-effectiveness of an orthopedic videoconferencing service between the University Hospital of North Norway and a regional medical center in a remote community located 148 km away.

**Methods:**

An economic evaluation based on a randomized controlled trial of 389 patients (559 consultations) referred to the hospital for an orthopedic outpatient consultation was conducted. The intervention group (199 patients) was randomized to receive video-assisted remote orthopedic consultations (302 consultations), while the control group (190 patients) received standard care in outpatient consultation at the hospital (257 consultations). A societal perspective was adopted for calculating costs. Health outcomes were measured as quality-adjusted life years (QALYs) gained. Resource use and health outcomes were collected alongside the trial at baseline and at 12 months follow-up using questionnaires, patient charts, and consultation records. These were valued using externally collected data on unit costs and QALY weights. An extended sensitivity analysis was conducted to address the robustness of the results.

**Results:**

This study showed that using videoconferencing for orthopedic consultations in the remote clinic costs less than standard outpatient consultations at the specialist hospital, as long as the total number of patient consultations exceeds 151 per year. For a total workload of 300 consultations per year, the annual cost savings amounted to €18,616. If costs were calculated from a health sector perspective, rather than a societal perspective, the number of consultations needed to break even was 183.

**Conclusions:**

This study showed that providing video-assisted orthopedic consultations to a remote clinic in Northern Norway, rather than having patients travel to the specialist hospital for consultations, is cost-effective from both a societal and health sector perspective. This conclusion holds as long as the activity exceeds 151 and 183 patient consultations per year, respectively.

**Trial Registration:**

ClinicalTrials.gov NCT00616837; https://clinicaltrials.gov/ct2/show/NCT00616837 (Archived by WebCite at http://www.webcitation.org/762dZPoKX)

## Introduction

Similar to many other countries’ publicly funded national health services, a key principle in Norway is that people should have equal access for equal need irrespective of their income or region of residence [1,2]. Thus, patients’ travel expenditures on public transportation are reimbursed, except a small user fee. In 2015, total reimbursement of patients’ travel expenditures accounted for 2.4% of the total budget for the specialist health services [3]. In particular, patients in the northern and western part of Norway have to travel long and often burdensome journeys to receive specialist care.

Musculoskeletal injuries are the most common causes of disability and chronic pain. Surgery for orthopedic conditions is witnessing some of the greatest growth rates in developed nations across the world [4]. Decentralizing orthopedic outpatient consultations is of special interest when a large number of patients live in remote areas, many of whom are not able to use public transport, or they need assistance by accompanying persons.

Decentralized services using outreach clinics or modern information and communication technologies have the potential to improve access, avoid patient travels, and reduce health sector costs. One such technology is telemedicine consultations using real-time videoconferencing. Today, the use of telemedicine to facilitate treatment and care over a distance has been investigated in almost all clinical specialties [5-7]. Several studies have demonstrated the feasibility of using telemedicine to provide orthopedic consultations to patients living in remote areas [8-11]. Teleorthopedics involve the delivery of specialist services across a distance, usually between an orthopedic surgeon and a patient [12]. It has been reported that teleorthopedics in an outpatient setting is safe and without serious adverse events [13], and that it has increased patient satisfaction [14,15], reduced travels and saved time for the patients [16,17], and reduced costs [18]. Teleorthopedics can also improve the effectiveness of rehabilitation after orthopedic surgery [4]. A study of pediatric orthopedic patients found that even greater benefit can be obtained from telemedicine consultation for patients with a disability where the cost and inconvenience of patient transport are considerably increased [19]. Videoconferencing has also successfully been used for distance training and educational purposes in the field of orthopedics [20,21]. Despite positive reporting of telemedicine studies, the uptake in clinical practice remains low [5,12,22,23].

There exist few randomized controlled trials (RCTs) evaluating telemedicine used in orthopedic outpatient clinics [24], and even fewer that have analyzed if teleorthopedic services are cost-effective compared with traditional outpatient consultations. The main arguments for introducing telemedicine services have been to reduce costs, improve efficiency, and increase quality of and access to health care services [5,25]. Hence, there is a need to determine the extent to which teleorthopedics proves to be cost-effective. Economic evaluation provides information about the costs and benefits of the alternatives under consideration [26]. Health care costs represent the value of resources used, such as staff, equipment, and consumables. Resources outside the health system can also be included such as the patients’ travel time and costs. Benefits refer to the value of changes in health outcomes. These changes can be negative and worsen health or positive and improve health [25].

In this study, we report the results from an economic evaluation. The primary objective of this study was to examine the cost-effectiveness of the telemedicine service, compared with standard in-person consultations at the hospital from a societal perspective. The secondary objective was to assess the robustness of the results by conducting sensitivity analysis. The costs included were health care costs, patient costs, and time costs measured as production loss. Health outcomes were measured as quality-adjusted life years (QALYs) gained.

## Methods

### Study Design and Recruitment

**Figure 1:**
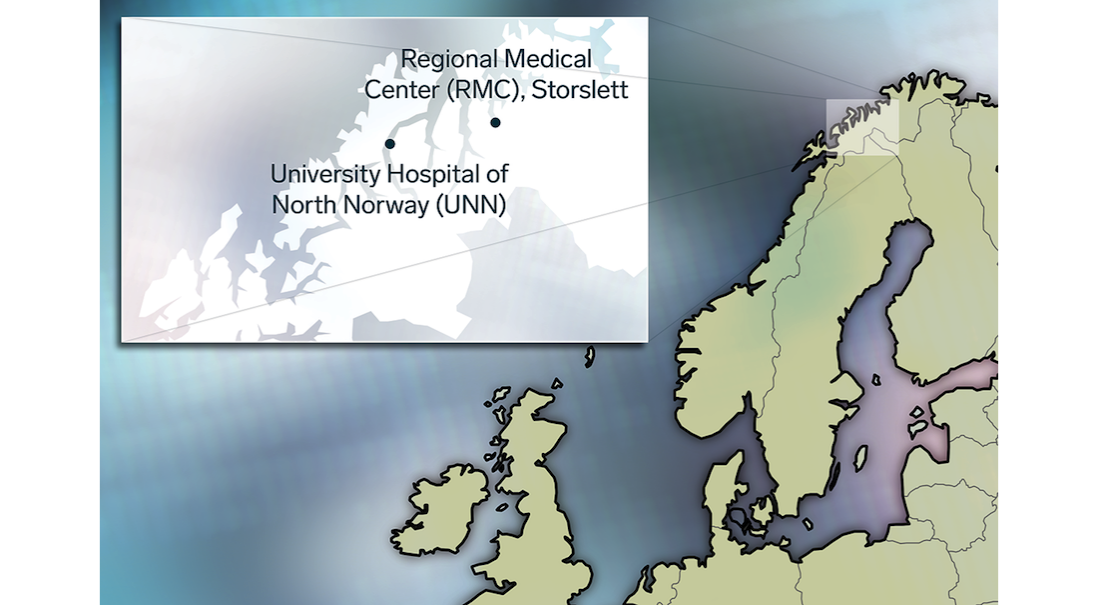
Norway and the area where the study patients were recruited and location of the University Hospital of North Norway and Regional Medical Center (inserted).

The economic study was based on a prospective RCT. This trial was conducted to assess if remote consultations using videoconferencing in orthopedic consultation was as safe and effective as in usual in-person care. Patients were recruited from the 4 northernmost municipalities in Troms County (Figure 1). All participants had been referred to or had scheduled a visit at the orthopedic outpatient clinic at the University Hospital of North Norway (UNN) and found to be suitable for a video-consultation. Patients were excluded if they were in need of any of the following: an advanced physical examination, a computed tomography scan, an ultrasound, an interpreter, seeing a specific surgeon, or if unable to give informed consent. Of the 402 patients who were randomized into 2 groups, 13 withdrew from the study or did not meet for the consultations. This left 389 patients in the study. Patients randomized to the intervention group received remote orthopedic video-consultations at the Regional Medical Center (RMC) (n=199). Patients randomized to the control group received standard outpatient consultations at the hospital (n=190). Informed consent was obtained from all the participants. The study was approved by the Regional Committee for Medical and Health Research Ethics.

### Equipment and Training

The remote consultations were performed through real-time videoconferencing. Both the remote center and the hospital used the Cisco TelePresence System and the Norwegian Health Network for secure data transmission (Screen: ViewSonic, Modl nr VS10946-Ie; at the remote center: Tandberg 990MXP; at the hospital: Tandberg 1500MXP). The orthopedic surgeons had some initial training and technical assistance in the beginning of the trial. Registered nurses were operating the service at the remote center. Before the trial, 2 nurses from the remote clinic received intensive training both to operate the technical equipment and to assist in treating the patients locally. They attended casting courses and were trained in clinical examination techniques.

### The Remote Consultation

The patients were scheduled for an appointment at the local center by the surgeon at the hospital. The orthopedic surgeons (3 consultants and 2 experienced registrars) were randomly selected to conduct the video-consultations if they were available at the specific time. The surgeon made the videoconference call to the remote center.

The patients showed up and were welcomed by one of the trained nurses who set up the videoconferencing at the remote site. The nurses assisted during the consultation and performed physical tasks, for example, changed a cast or removed stitches. No physician was present during the video consultations at the remote site. An existing digital X-ray lab served by a local radiographer was available at the remote clinic. Digital X-rays were, if needed, available and shown to the patients at the time of the consultation.

### Usual Care

In the control group, patients received standard consultations at the hospital outpatient clinic. In 32% of the standard consultations, the orthopedic surgeons needed assistance from a nurse [13].

In both the standard and the video consultation alternatives, the usual mandatory registration and documentation in patients’ medical records were carried out by the orthopedic surgeon. This involves the conclusion of the consultation, agreement between surgeon and patient regarding any follow-up appointments, prescriptions, referrals for operation, further investigations, physiotherapy, and an application for orthopedic aid if needed. The average number of consultations per patient was 1.5 (range 1-6). For more details of the trial method, see Buvik et al [13].

### Economic Evaluation

The economic evaluation consisted of trial-based analyses following the guidelines for health economic evaluation [27,28]. The cost and effectiveness data used in the economic study were based on actual investments, personnel costs, patient travels, and health outcomes collected during the trial described above. A societal perspective was adopted for calculating costs including health care costs, private costs, and production loss. Effectiveness was measured in terms of QALYs gained.

Data on costs and QALYs gained were collected alongside the trial at baseline and at 12 months follow-up using questionnaires, patient charts, and consultation records. These were valued using externally collected data on unit costs and utilities. To increase generalizability and make the cost-effectiveness result useful for decision making, the resources used in the trial were valued using equipment prices, unit costs, travel fares, and salaries from 2017/2018. An extended sensitivity analysis was conducted to address the robustness of the results.

### Costs

Three types of costs were included: (1) costs associated with implementing and running the telemedicine service in clinical practice, (2) travel costs, and (3) production losses.

#### Costs Associated With Implementing and Running the Service

The implementation costs included the costs related to the investment in videoconferencing equipment (codec, screen, and camera) at the remote center and the hospital, and an extra computer and printer at the remote center. The remote center already had a broadband connection for other purposes. The computer at the remote center provided the nurse access to the patients’ hospital records, and the printer was used to give patients a paper copy of the records on request. In addition, costs related to running the service at the remote center were estimated. This included a registered nurse in a 20% position. Other costs associated with setting up the service included initial training sessions and travel costs related to these activities, line rent, and rent associated with the extra space needed at the local center. No extra technical support was needed as they used existing resources at the hospital. The costs estimated were only those that differed between telemedicine and hospital consultations, that is, the incremental costs. The time costs for the orthopedic surgeons were the same for both consultation forms [13]. The consumables, X-ray, and administrative costs were assumed to be similar for both groups. The cost of the nursing assistance during the standard consultations was also included. Equipment prices and line rent for both the remote clinic and the hospital were collected from the purchasing department at the hospital. A one-time equipment cost can be spread over the expected lifetime of the equipment by annuitizing the cost using a discount factor. The investment costs including equipment, installation, and training were annuitized into an equivalent annual cost assuming a 3% discount rate and a 5-year lifespan for the equipment. The costs of the extra space and other facilities at the local center were collected from financial and administrative records at the hospital. Official salary for nurses was used to estimate the costs of the extra nurse position. The costs are presented as total annual costs and costs per patient consultation (unit costs).

#### Travel Costs

Travel costs were collected directly from the patients during the trial. Data on traveling time, distance, and mode of transport to the consultation were collected using a questionnaire that was handed to the patient directly after each consultation. Main occupation, if they were on sick leave, and the need of overnight stay were also included. Additionally, Google map was used to estimate the travel distance from the patients’ home to the consultation site either at the remote center or at the hospital (shortest and fastest). The orthopedic surgeon decided if the patient needed a companion or extra transportation on health-related grounds, reported the patients’ main occupation, and if they needed sick leave. The travel costs were calculated using regulations and official travel fare rates by the Norwegian Patient Travel Agency in 2018 [29].

#### Production Losses

Production losses were estimated for patients in full- or part-time employment who had to take time off from work to attend the orthopedic consultations. Part-time employment was set to 50%. Time costs for the patients who were unemployed or on sick leave benefits were not included. If the information about working status was missing from the self-reported questionnaires, the orthopedic surgeons’ registration forms were used. Official Norwegian average wages were used to value absence from work to estimate the production losses.

Only 3 of the 199 patients who were offered a video consultation at the remote center had a new consultation at the hospital, because of their need for a face-to-face consultation to carry out examination that is not possible over the video link. The cost of these second consultations was also included in the analysis. Based on the resources available at the hospital and the experiences from the trial, we assumed that for 300 patient consultations annually, 5 would need a second consultation.

### Quality-Adjusted Life Years Gained

Health outcomes were measured in QALYs gained, a composite measure incorporating both quantity and quality-of-life impacts of treatment [26]. As a patient-reported outcome measure, we applied the EQ-5D which is the most widely used generic preference-based instrument for valuing QALYs [30]. EQ-5D questionnaires were collected at baseline and at 12 months follow-up. The questionnaires were handed to the patients immediately after the first consultation and sent by mail 12 months after the last consultation during the trial. The scoring algorithm estimated for a sample of the general population in United Kingdom was used to calculate utility values from the utility scored in the EQ-5D instrument (the EuroQol health states) [31]. Utility values were calculated only if all 5 of the EQ-5D dimensions were answered. Finally, QALYs were then calculated by multiplying the change in utility value with the duration of the health state (1 year) [26].

### Statistical Analysis

Results are presented as means (SDs) or numbers (percentages). Differences between the groups were analyzed using 2 sample *t* tests, chi-square tests, or generalized estimating equations (GEEs). GEEs were used with an exchangeable covariance structure to control for dependence between 2 or more repeated consultations for some participants. All statistical analyses were performed using STATA version 14.0 (StataCorp LP Texas, USA).

### Sensitivity Analysis

One-way sensitivity analyses were conducted to assess the robustness of the results. Parameters have been varied one at a time to assess the effect on the cost-effectiveness and to determine breakeven values. We recalculated the cost analysis in 3 separate scenarios: one included a less costly Skype for Business solution, the second assumed a shorter distance to the main hospital, and a third scenario includes the expenditure to the hospital excluding patients’ own travel expenditures and production losses. The number of consultations needed to break even was calculated for all scenarios.

## Results

Table 1 provides characteristics of the participants at baseline.

**Table 1:** Descriptive baseline characteristics from first consultation according to location.

Baseline characteristics of the participants (n)		UNN^a^ standard consultation (n=190)	RMC^b^ telemedicine consultation (n=199)
Males, n (%)		75 (39.5)	82 (41.2)
Age (years), mean (SD)		46.7 (24.9)	48.8 (24.0)
**Age group (years), n (%)**			
	1-15	42 (22.1)	29 (14.6)
	16-66	94 (49.5)	117 (58.8)
	67-90	54 (28.4)	53 (26.6)
**Patient residential municipality, n (%)**			
	Kvænangen	25 (13.2)	26 (13.0)
	Nordreisa	82 (43.2)	90 (45.2)
	Skjervøy	47 (24.7)	45 (22.6)
	Kåfjord	36 (18.9)	38 (19.1)
**Cause of consultation, n (%)**			
	New referral	69 (36.3)	81 (40.7)
	Control after elective surgery	25 (13.2)	22 (11.1)
	Control after trauma, surgery	33 (17.4)	35 (17.6)
	Control after trauma, no surgery	55 (28.9)	50 (25.1)
	Chronic disease	8 (4.2)	11 (5.5)
**Employment status (n=177+190)^c^, n (%)**			
	Full-time worker	45 (25.4)	56 (29.5)
	Part-time worker	23 (13.0)	20 (10.5)
	Homemaker	12 (6.8)	19 (10.0)
	Unemployed	2 (1.1)	2 (1.1)
	Retired or disability benefit	55 (31.1)	61 (32.1)
	Student or pupil	40 (22.6)	32 (16.8)
EQ-5D-3L index (n=165+178)^c^, mean (SD)		0.70 (0.25)	0.68 (0.26)
EQ VAS 1-100 (n=140+150)^c^, mean (SD)		75 (18)	73 (19)

^a^UNN: University Hospital of North Norway

^b^RMC: Regional Medical Center.

^c^Number of item responses in UNN and RMC, respectively.

### Costs

#### Costs Associated With Implementing and Running the Service

The costs of setting up the teleorthopedic service are presented in Table 2. Total costs of investing in standard videoconferencing units at both sites were €16,511 (1 Euro=9.60 Norwegian krone, April 10, 2018). The total annual costs including annuitized investment costs (equipment and initial training), line rent, extra personnel costs, and rent for extra office space at the regional center were €20,684. The largest cost component is the extra nursing costs at the local health center. Nearly, two-thirds of the total annual cost of the teleorthopedic service are extra personnel costs. If a less costly Skype for Business alternative had been used, the annual costs would have been reduced to €17,535 (see Table 2 for more details).

**Table 2:** The costs of setting up a video-assisted outpatient clinic (in Euro).

Cost elements		UNN^a^ standard consultation	RMC^b^ telemedicine consultation	Total (Euro)^c^	Annual cost
**Alternative A^d^**					
	Videoconferencing equipment	5104	6250	11,354	—
	Personal computer (PC)	—	463	463	—
	Screen	—	156	156	—
	Printer	—	114	114	—
	Initial training of nurse and physician	—	—	4424	—
Total investment A				16,511	3605^e^
**Additional costs alternative A**					
	Line rental, Norwegian Health Net	—	104^f^	—	1250
Sum alternative A					4855
**Alternative B^g^**					
	Camera	96	937	1033	—
	Screen	—	833×2	1666	—
	Microphone	111	—	111	—
	PC	—	463	463	—
	Printer	—	114	114	—
	Initial training of nurse and physician	—	—	4424	—
Total investment B				7811	1706^e^
**Additional costs alternative A and B**					
	Technical support^h^	—	—	—	—
	Rent for local RMC	—	—	—	3542
	Nurse at RMC^i^	—	—	—	12,083
	In need of a second consultation at the hospital^j^				204
Total additional costs					15,829
Total annual cost alternative A					20,684
Total annual cost alternative B					17,535

^a^RMC: Regional Medical Center, remote location.

^b^UNN: University Hospital of North Norway, standard consultation.

^c^1 Euro=9.60 Norwegian krone, exchange rate from the Norwegian Bank on April 10, 2018.

^d^Alternative A: Videoconferencing units: UNN—Cisco TelePresence System EX90; RMC—Cisco TelePresence MX200 G2 (prices obtained from the purchasing department at the hospital).

^e^Annual cost has been calculated using a 3% discount factor and a 5-year lifetime of the equipment.

^f^Per month.

^g^Alternative B: Skype for Business: UNN—Camera Logitech: Webcam C930e—net camera; Tablemicrofon: Jabra SPEAK 510+MS (already installed 1 PC and 2 screens for standard consultations); RMC—2 screens Philips Signage Solutions Q-Line BDL5535QL+camera/microphone Logitech GROUP+PC (prices obtained from the purchasing department at the hospital).

^h^Technical support—no extra costs included as this support has been covered by existing support at the hospital.

^i^20% part time, including social costs.

^j^Three patients needed a second consultation at UNN because of an unsatisfactory consultation at the RMC during the trial (out of 199 patients) [13]. Since we have assumed 300 patients a year in the teleconsultation alternative, costs of a second consultation have been included for 5 patients per year.

#### Travel Costs

Table 3 shows details on patients’ modes of transport. Most patients in the remote group traveled by private car. The chosen mode of transportation reflects the lack of available public transportation in the area. In the group of patients traveling to the hospital, 26% needed extra transportation facilities because of their health condition. This number was 30% for the patients in the telemedicine group (*P*=.31). In addition, the need for travel companions was the same in both groups (30% in the hospital consultation group and 27% in the telemedicine group; *P*=.45). The time spent on traveling was 6 times higher for patients traveling to the hospital. Patients in the telemedicine group saved an average 7 hours and 40 minutes on traveling (see Table 4 for more details). The average travel cost per patient is €148.65 for the standard consultations at the hospital, as compared with €40.73 for the video consultations, including user fees for the patients (€31.04; see Multimedia Appendix 1).

**Table 3:** Patient transport mode to each consultation per allocation. Of the 389 patients participating in this study, some attended more than 1 consultation; consequently, the total number of consultations in this study was 559 (257 at UNN and 302 at RMC).

Transport mode	UNN^a^, n (%)	RMC^b^, n (%)	*P* value^c^	*P* value, GEE^d^
Taxi^e^	55 (21.4)	60 (19.9)	.57	.77
Taxi, as main transport	47 (18.3)	60 (19.9)	.71	.56
Airplane	3 (1.1)	0	N/A^f^	N/A
Bus^g^	72 (28.0)	6 (2.0)	<.001	<.001
Bus, as main transport	66 (25.7)	6 (2.0)	<.001	<.001
Private car^h^	106 (41.2)	211 (69.9)	<.001	<.001
Private car, as main transport	98 (38.1)	209 (69.2)	<.001	<.001
Express boat	28 (10.9)	0	<.001	N/A
Ferry^i^	19 (7.4)	7 (2.3)	.004	.01
Other^j^	0	15 (5.0)	<.001	N/A
Not reported or Missing	16 (6.2)	13 (4.3)	.31	N/A

^a^UNN: University Hospital, standard consultation.

^b^RMC: Regional Medical Center, remote location.

^c^Test for equality between UNN and RMC using chi-square test.

^d^Test for equality between UNN and RMC using generalized estimating equations (GEEs) with a logit link function and a binary response, transport (yes or no).

^e^Including taxi as shuttle to other transport (bus, express boat, or airplane).

^f^N/A: not applicable, few or no observations.

^g^Including bus as shuttle to other transport (airplane or express boat).

^h^Including private car as shuttle to other transport (bus, express boat, airplane, or taxi).

^i^Always in combination with other transport (bus, private car, express boat, or taxi).

^j^Walking, bicycle, or working car.

**Table 4:** Patients’ travel details and working status.

Patients’ travel and working status	UNN^a^ standard consultation	RMC^b^ telemedicine consultation	*P* value^c^
Travel distance in kilometers, shortest distance^d^ (n=257+302)^e^, mean (SD)	148 (31)	46 (17)	<.001
Travel distance one way in kilometers^f^ (n=224+284)^e^, mean (SD)	248 (59)	47 (28)	<.001
Travel time one way in minutes^f^ (n=243+293)^e^, mean (SD)	277 (94)	47 (43)	<.001
Need of companion, (n=245+294)^e^, n (%)	73 (29.8)	79 (26.9)	.45
Need of extra transport^g^, (n=249+297)^e^, n (%)	64 (25.7)	88 (29.7)	.31
Working full time^h^, (n=136+179)^i^, n (%)	59 (43.4)	75 (41.9)	.68
Working part time^h^, (n=136+179)^i^, n (%)	29 (21.3)	28 (15.6)	.68
Sick leave—all^j^, (n=138+180)^i^, n (%)	60 (43.5)	71 (39.4)	.47
Actual working^k^—full time, (n=76+109)^i^, n (%)	20 (26.3)	36 (33.0)	.09
Actual working^k^—part time, (n=76+109)^i^, n (%)	15 (19.7)	13 (11.9)	.09

^a^UNN: University Hospital North Norway, standard consultation.

^b^RMC: Regional Medical Center, remote location.

^c^Test for equality between UNN and RMC using *t* test or chi-square test as appropriate.

^d^Calculated road between allocation and municipality center using Google Map, one way. The travel distance for the patient in the municipality, where the RMC is located, is replaced with the mean value of the municipalities’ patients reported travel distance.

^e^Number of item responses in UNN and RMC, respectively.

^f^Patients reported distance or time used to travel to the consultation.

^g^Need extra transport, as patient was not able to use public transport.

^h^Patient reported (age between 15 and 67 years), missing value adjusted by doctors reported value.

^i^Number of item responses in UNN and RMC, respectively, age between 15 and 67 years.

^j^Including unemployed and homemakers.

^k^Working—patient not with sick leave.

#### Production Losses

Production losses for patients who had to be away from work to attend the consultations, the total average costs of the patient transfer amounted to €182.50 per patient for the standard consultations and €51.77 for the teleconsultations. The calculation of travel and time costs for the patients is presented in detail in Multimedia Appendix 1.

### Quality-Adjusted Life Years Gained

The average QALYs gained per patient in the telemedicine group was .09 which was not significantly different to the .05 gain in the standard consultation group, *P*=.29.

### Cost and Effectiveness

**Figure 2:**
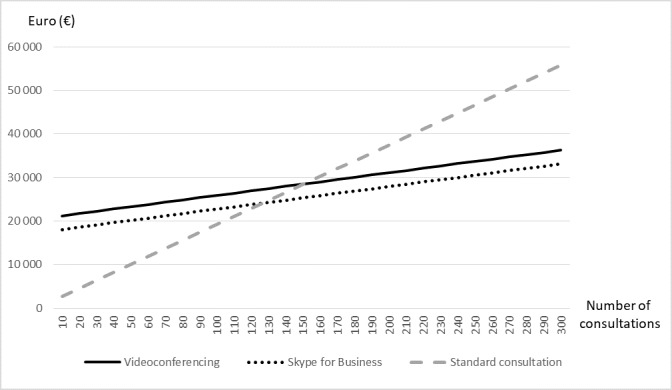
Total annual costs of the teleorthopedic service including the Skype for Business alternative.

Table 5 presents the costs and effects in each of the 2 alternatives. Among patients in the intervention group, 3 needed a second face-to-face consultation that was not possible to carry out over the video link. The cost of these second consultations was also included in the analysis (Table 5). In total, the telemedicine service costs €65 less per patient than standard consultations at the hospital. Thus, the remote teleorthopedic service is less costly and produced no difference in health outcome, that is, the teleorthopedic service as described in this study is cost-effective. The number of patient consultations needed for telemedicine and standard consultation to be equally costly (breakeven) is 151 patients per year (see Figure 2).

**Table 5:** Costs and effectiveness for standard and remote consultations (1 Euro=9.60 Norwegian krone, exchange rate from the Norwegian Bank on April 10, 2018).

Costs and effectiveness		UNN^a^ standard consultation	RMC^b^ telemedicine consultation	Difference
**Consultation costs^c^ (Euro)**				
	Investment cost videoconferencing^d^	0	3605	3605
	Line rent	0	1250	1250
	Room rent	0	3542	3542
	Personnel costs (nurse)^e^	906	12,083	11,177
	In need of a second consultation at the hospital^f^	0	204	204
Total annual costs		906	20,684	19,778
Cost per consultation^g^		3	69	66
**Time and travel costs (Euro)**				
	Travel costs	149	41	108
	Time costs^h^	34	11	27
Total time and travel costs per consultation		183	52	131
Total costs per patient consultation		186	121	65
Effectiveness (QALYs^i^ gained)		.05	.09	.04^j^

^a^UNN: University Hospital of North Norway.

^b^RMC: Regional Medical Center.

^c^Consultations cost which are different between the 2 groups.

^d^Total investment costs have been annuitized using 3% discount factor and a 5-year lifetime.

^e^The extra personnel costs at the remote location included a nurse in 20% position. At the standard consultation, a nurse was present in 32% of the consultations, corresponding to 25 hours by 300 consultations a year.

^f^Three patients needed a second consultation at UNN because of an unsatisfactory consultation at the RMC during the trial (out of 199 patients) [13]. Since we have assumed 300 patients a year in the teleconsultation alternative, costs of a 2^nd^ consultation have been included for 5 patients per year.

^g^The annual load for this service is estimated to be 300 telemedicine consultations per year.

^h^Production loss because of absence from work to receive orthopedic consultation.

^i^QALYs: quality-adjusted life years.

^j^The difference in QALYs gained was not significant (*P*=.29) *t* test.

### Sensitivity Analysis

**Figure 3:**
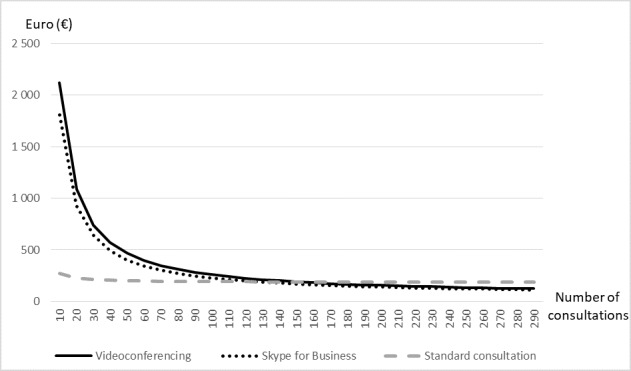
Cost per patient for base case and the Skype for Business alternative.

The different scenarios in the sensitivity analyses are illustrated in Figures 2 and 3. The main case alternative described above is represented by the solid black curve (videoconferencing) and the gray dotted line (standard consultation at the hospital). The first scenario included a less costly Skype for Business alternative. The total annual cost of this alternative was €17,535, and the number of patient consultations needed to break even was 127 per year (dotted black line). The second scenario, assuming a shorter distance (90 km) between the remote clinic and the hospital, needed 314 patient consultations to breakeven using videoconferencing units (not shown). The third scenario included only expenditures to the hospital excluding production losses and patients’ own travel expenditures. This made telemedicine cost-effective for an activity of at least 183 patient consultations a year (not shown).

## Discussion

### Principal Findings

The results of this study showed that using videoconferencing to offer orthopedic consultations to patients at the remote clinic costs less than standard outpatient consultations at the specialist hospital, as long as the activity exceeds a minimum of 151 patient consultations per year. For a total workload of 300 patients per year, the annual cost savings amounted to €19,500. With a health care sector cost perspective, the number of patient consultations needed to break even was 183, and the total annual savings amounted to €12,600. Thus, teleorthopedics is cost-effective from both a societal and health care provider perspective. A shorter distance to the hospital was the only scenario that altered the conclusion. Reducing the travel distance by 50 km made the standard consultation more cost effective for up to 314 patients per year.

Assuming a less expensive Skype for Business alternative reduced the cost of telemedicine with €3149 annually, this alternative became cost-effective when including more than 127 patient consultations per year. The reduction in costs by investing in a cheaper videoconferencing solution was relatively modest. One of the reasons is that the equipment cost was less compared with the other cost components such as the extra personnel needed at the remote site. The quality of the videoconferencing might also be reduced using Skype for Business, and more patients would need a second consultation at the hospital making the cost advantage even less. We have not evaluated if a Skype for Business alternative will reduce the quality in picture and/or sound transmission. Other options to reduce the equipment costs are to increase utilization and share the videoconferencing units with other specialties/other use (eg, teaching or meetings) [32]. These possibilities should be considered before setting up video consultations in a specific field.

In most of the published literature, a physician (eg, a general practitioner [GP], a general surgeon, or a resident) has been present at the remote site together with the patient [8,10,11,33,34]. Some studies reported that a nurse at the remote site and a specialist at the hospital could provide satisfactory remote consultations in emergency medicine [35-37]. Wallace et al recommend to include a nurse to host the teleconsultations in place of the GP to reduce the cost of telemedicine [38]. In this study, a nurse was hosting the teleconsultations at the remote site. To our knowledge, no other studies have reported a similar setting when studying the use of teleorthopedics for newly referred patients or follow-up consultations. However, the extra personnel costs still consist of nearly two-thirds of the total annual cost of the teleorthopedic service even if a nurse is hosting the teleconsultations.

Whether to include production losses measured as time off work is controversial [39]. The patients may already be off work, because they are retired or because of their health condition. Health visits of a shorter duration might not represent production losses at all. Some types of work can be postponed until the person is back or one’s colleagues can take over. The time costs are important in telemedicine and eHealth, and one should find a way to include these costs [25]. In this study, production loss has only been included for those who reported that they took time off work to attend the consultation. From a societal perspective, these costs are relevant, but not from a health care provider perspective. Excluding the production loss does not change the cost-effectiveness as shown in the sensitivity analysis. Another way is to report the time (hours or days) lost or gained separately without putting a value on it [26]. Our results show that patients receiving standard consultations spent almost 8 hours more per consultation traveling than the patients in the telemedicine group.

Other studies have reported a reduction in the number of referrals to the specialist because of a learning effect and included this as part of the cost savings [32,40]. In this study, the nurse at the remote center reported an increase in the number of patients treated locally mostly because of their newly acquired casting skills. This was seen for patients with a stable fracture (not displaced). If this effect had been included in our analysis, the service would have reduced the need for specialist referrals and incurred additional cost savings.

A third option to prevent the patient from traveling long distances is to have the specialist travel from the hospital to the remote location. However, because of a lack of orthopedic specialists at the central hospital in this region, the opportunity costs of their travel time would be too high. However, in other institutional contexts, this might be another alternative to consider.

In this study, we demonstrated that significantly less public transport such as a bus or boat was used in the remote consultation group (Table 3). This can be explained by the lack of available public transport in the rural area, something that explains the frequent use of taxi and private cars. Expensive and long travels imply that fewer patients are needed to make remote consultations cost-effective.

### Strengths and Limitations

The main strength of this study is that the costs and effects have been collected alongside an RCT. The estimated travel costs included in the analysis are based on the actual journeys undertaken by the participants in the trial. The time and travel cost calculation was based on actual travel distances, modes of transportation, how many in need of a companion, time spent on traveling, and the working status of each patient. Official travel fares reimbursed from the Norwegian Patient Travel Agency were used as unit costs. Some of the patients’ journeys were organized by the Norwegian Patient Travel Agency (most of the taxi trips and flights). The patients had to apply for reimbursement for additional expenses.

Official travel fares reimbursed by the Norwegian Patient Travel Agency were used to calculate the travel costs. If the patients chose to travel by a more expensive alternative than the travel agency’s reimbursement (eg, by plane), the patients had to pay for the difference themselves. This makes the true travel costs for the patients potentially higher than estimated in this study. It is also possible that the actual travel costs for the health care sector are lower than calculated. Some patients did not apply for travel refund, either because they forgot or simply because they found it too troublesome to fill out the forms. One study from Norway demonstrated that 26% of the patients and 70% of the accompaniers did not apply for a travel refund [41].

Another limitation of this study is that production loss for the persons accompanying the patients on travels was not included. The main reason for this was the lack of information about their working status, and it was considered important to avoid overestimating the benefits of the service. About 28% of the patients needed a travel companion. If these time costs were included, it would have made the teleorthopedic service even more cost-effective.

Training costs have been included as a one-time cost at the startup of the teleorthopedic service. However, training should be included as an ongoing part of the service to promote and sustain use. Training is necessary because of staff turnover, particularly at the hospital. Casting courses and training in clinical examination techniques must also be arranged if there is a change in the nursing staff locally. Telemedicine services are often sensitive to changes in key personnel [42]. Successfully sustaining telemedicine services is about integration and effective change management [43].

### Generalizability

One challenge for economic evaluations of telemedicine services is generalizability. High diversity in terms of objectives, technology, application, and context might limit the generalizability of specific evaluations to other settings [44]. The local context will decide the cost parameters such as the need for investment in technology and infrastructure, prices, the costs of extra personnel, and travel and time costs. The results of an economic evaluation are of most value for decision makers in the local area where the evaluation was conducted. It is important to assess if the assumptions, cost parameters, and context can be compared between settings.

To make this study relevant outside of the current institutional setting, we have emphasized a transparent calculation of all cost and effectiveness items, based on 2 costing perspectives. The same methodology can then be generalized, however, based on local prices.

### Conclusions

This study showed that providing video-assisted orthopedic consultations at a remote clinic, rather than having patients travel to a centrally located hospital for consultations is cost saving. This conclusion holds from both a societal and a health sector perspective and as long as the activity exceeds 151 and 183 patient consultations per year, respectively.

## Appendix

Multimedia Appendix 1 Calculation of patients cost for videoconferencing and standard consultations (in Norwegian kroner and Euro).

## Abbreviations

GEE: generalized estimating equation
GP: general practitioner
QALY: quality-adjusted life year
RCT: randomized controlled trial
RMC: Regional Medical Center
UNN: University Hospital of North Norway
