# Supplementary material for: Cost-Effectiveness of Telemedicine in Remote Orthopedic Consultations: Randomized Controlled Trial
Source: J Med Internet Res. 2019 Feb 19;21(2):e11330. doi: 10.2196/11330 (PMC6399572; doi:10.2196/11330)
Supplement: Multimedia Appendix 1 [file jmir_v21i2e11330_app1.pdf]

Supplementary Table 6 Calculation of patients cost for videoconferencing and standard consultations (in Norwegian krone and Euro[a])

	UNN					RMC				
	n 257	Mean distance/ number/time	Fare price/ unite price, NOK	Total costs	Total costs per patient	n 302	Mean distance /time	Fare price/ unite price, NOK	Total Costs	Total costs per patient
**Travel costs without companion**	**184**					**223**				
Using standard rate[b]	114	x 144km[c]	x 2.40 x 2	78,797		143	x 44km[c]	x 2.40 x 2	30,202	
Extra transport – private car	12	x 254km[d]	x 2.40 x 2	14,630		28	x 45km[d]	x 2.40 x 2	6,048	
Taxi –round trip	35	x 227km[e]	x 12.50[f]	99,313		52	x 52km[e]	x 16.50[f]	44,616	
Taxi- waiting		x 4 h	x 190[f]	26,600			x 1 h	x 253[f]	13,156	
Airplane	2		x 713 x2	2,852						
Express boat	21		x 430 x 2	18,060						
Overnight stay; >12 h[g]	10		x 220	2,200		2		x 220	440	
**Travel costs with companion**	**73**					**79**				
Using standard rate[b]	38	x 151km	x 2,40 x 2	27,542		59	x 45km[c]	x 2.40 x 2	12,744	
Extra transport – private car[b]	4	x 275km[c]	x 2.40 x 2	5,280		12	x 46km	x 2.40 x 2	2,650	
Bus	13									
Kvænangen	4	x 212km[c]	x 2.40 x 4	8,141						
Nordreisa	7	x 140km[c]	x 2.40 x 4	9,408						
Skjervøy	1	x 160km[c]	x 2.40 x 4	1,536						
Kåfjord	1	x 113km[c]	x 2.40 x 4	1,085						
Taxi – round trip	12	x 226km[e]	x 16.50[f]	44,748		8	x 46km	x 16.50[f]	6,072	
Taxi - waiting		x 4 h	x 253[f]	12,144			x 1 h	x 253[f]	2,024	
Airplane	1		x 713 x 4	2,852						
Express boat	5		x 430 x 4	8,600						
Overnight stay; >12 h[g]	7		x 220 x 2	3,080						
**Total travel costs,** NOK(**€)**				**366,868**	**1,427 (148.65)**				**117,952**	**391 (40.73)**
Patient's charge for the journey, NOK(**€)**			149 x 2		298(31.04)			149 x 2		298(31.04)
**Total travel cost health system,** NOK(**€)**					**1,129 (117.60)**					**93(9.69)**
**Time costs**										
Lost working hours - full time[h]	20	x 8h	x 380[i]	60,800		36	x 0.93h x 2	x 380[i]	25,445	
Lost working hours - part time[h]	15	x 4h	x 380	22,800		13	x 0.65h x 2	x 380	6,422	
**Lost working hours total,** NOK(**€)**				**83,600**	**325 (33.85)**				**31,867**	**106 (11.04)**
**Total travel costs and lost working hours for society, NOK (€)**					**1,752 (182.50)**					**497 (51.77)**

UNN – University Hospital, standard consultation; RMC – Regional medical center, remote location; n= number of traveling to consultation, missing value is replaced with lowest estimated value/distance/time according to travel-distance.

[a] 1 Euro = 9.60 Norwegian krone (NOK), Exchange rate from the Norwegian Bank on April 10[th]2018

[b] Standard rate refunded 2.40 NOK per kilometer according to Pasientreiser HF (the National Patients Transport) for 2018. Non responder is included as standard rate refunded

[c] Mean distance (the shortest distance)

[d] Mean distance (patient reported distance)

[e] Mean distance (the shortest and fastest - no ferry)

[f] Adjusted for estimated number of patient in the same taxi, reported from the UNN's Patient Travelling Agency

[g] Stay overnight, refunding 220 NOK (undocumented) subsistence allowance,

[h] Directly waiting time before consultation and consultant time is not included. The difference between the location in pre-consultation waiting time is small (7 minute longer at UNN, p-value <0.001). There was no significant difference in mean consultation time (20.9 and 20.5 minutes)

[i] Average income all sectors per hour, 150 hours per week, 2017 + 30% social taxes according: https://www.ssb.no/en/arbeid-og-lonn/statistikker/lonnansatt
